# Impact of a Multimodal Prehabilitation Program on Perioperative Outcomes in Hepatopancreatobiliary Surgery: A Retrospective Cohort Study

**DOI:** 10.3390/curroncol33040207

**Published:** 2026-04-05

**Authors:** Pipit Burasakarn, Nattaporn Maneepairoj, Vachiraluck Chalokool, Anuparp Thienhiran, Sermsak Hongjinda, Pusit Fuengfoo

**Affiliations:** Division of HPB Surgery, Department of Surgery, Phramongkutklao Hospital, Thung Phaya Thai, Ratchathewi, Bangkok 10400, Thailand

**Keywords:** prehabilitation, hepatopancreatobiliary surgery, hepatectomy, pancreatectomy, perioperative outcomes

## Abstract

**Background**: Major hepatopancreatobiliary (HPB) surgeries cause significant physical stress. In this study, we evaluated how a 4-week multimodal prehabilitation program affects perioperative outcomes across different types of HPB procedures. **Methods**: We conducted a retrospective cohort study of 359 patients undergoing HPB surgery (162 historical controls and 197 in the prehabilitation group). To accurately assess the clinical benefits of various procedures, patients were stratified into specific surgical groups: major and minor hepatectomy, pancreatoduodenectomy (PD), and left pancreatectomy (LP). **Results**: The prehabilitation program significantly improved preoperative nutrition, demonstrated by increased serum albumin levels on the day of surgery (*p* < 0.001), and the clinical benefits were most pronounced in pancreatic surgeries. Patients undergoing PD in the prehabilitation group had a significantly shorter median hospital stay (8 versus 13 days, *p* < 0.001). LP patients also experienced shorter hospital stays (5 versus 9 days, *p* = 0.001) and reduced blood loss (*p* = 0.002). For minor hepatectomies, the intervention significantly lowered the need for blood transfusions (8.3% versus 18.9%, *p* = 0.033). The length of stay and complication rates for major hepatectomies remained comparable between groups. Importantly, major morbidities and 90-day mortality were low and similar across all cohorts. **Conclusions**: Multimodal prehabilitation successfully optimizes preoperative nutrition and accelerates hospital discharge, especially after highly stressful pancreatic surgeries. Because benefits vary by surgical magnitude, prehabilitation pathways should be tailored to prioritize high-risk patients facing complex operations.

## Simple Summary

Major surgeries involving the liver and pancreas are physically demanding and often lead to long hospital stays. This study investigated whether a four-week “prehabilitation” program—consisting of supervised nutrition, exercise, and mental health support—could better prepare patients for these operations. We found that patients who participated in the program had better nutritional health on the day of surgery. Most importantly, patients undergoing complex pancreatic procedures recovered significantly faster, leaving the hospital up to five days earlier than those who did not participate. While the benefits were most noticeable in the most difficult surgeries, the results suggest that strengthening a patient’s physical reserve before an operation can lead to a quicker and more efficient recovery. These findings support making personalized “fitness for surgery” programs a standard part of hospital care.

## 1. Introduction

Major abdominal procedures, particularly hepatopancreatobiliary (HPB) resections, are among the most technically challenging operations in gastrointestinal surgery. Despite notable progress in surgical techniques, anesthesia, and perioperative management, patients undergoing extensive HPB resections continue to face considerable postoperative risk. Multicenter benchmarking studies have reported major morbidity rates between 20% and over 30%, with rates largely dependent on the complexity of the resection [1,2,3]. Additionally, recent publications highlight that 90-day mortality now serves as a more accurate measure of patient outcomes than 30-day mortality, given its superior ability to capture delayed complications and rescue efforts from severe physiological insults [4]. The significant physiological stress resulting from major HPB surgery leads to systemic inflammatory response, catabolism, and predictable reductions in functional capacity. While Enhanced Recovery After Surgery (ERAS) protocols have successfully standardized postoperative care and reduced hospital stays, their primary focus is on minimizing stress during and following the surgical event. ERAS protocols alone may not adequately address the diminished baseline physiological reserve observed in many HPB patients, especially those presenting with preoperative weight loss, sarcopenia, or jaundice secondary to malignancy. This limitation has prompted a shift toward preoperative optimization, aiming to better prepare patients both physically and mentally for forthcoming surgical trauma. Prehabilitation has been recognized as an essential proactive intervention to counteract expected physiological decline. Defined as the enhancement of functional capacity prior to scheduled physiological stress, multimodal prehabilitation generally includes targeted exercise, nutritional optimization, and psychological support. Exploiting the pre-surgical waiting period, this approach strives to build a robust “physiological buffer”. The principal aim is not simply to improve baseline fitness but also to bolster resilience, thereby reducing postoperative complication severity and facilitating quicker recovery to baseline function [5,6,7,8]. Although there is evidence to support the benefits of prehabilitation in general abdominal and colorectal surgery, its specific effects within the diverse HPB patient population remain to be fully elucidated. Furthermore, it is unclear whether intensive prehabilitation programs yield consistent clinical benefits across all HPB procedures, from minor hepatectomies to complex pancreatoduodenectomies. Accordingly, this retrospective cohort study evaluates the impact of a multimodal prehabilitation program on perioperative outcomes in HPB surgery at a high-volume tertiary referral center, with attention to outcome stratification by the extent and type of surgical resection The present study aims to assess the influence of prehabilitation on perioperative outcomes in hepatobiliary and pancreatic surgery, with particular emphasis on postoperative complications, morbidity, and 90-day mortality.

## 2. Methods

### 2.1. Study Design and Ethics

This study was conducted at Phramongkutklao Hospital, a high-volume tertiary referral University hospital in Bangkok, Thailand. The study methodology and reporting were conducted in strict adherence to the Strengthening the Reporting of Observational Studies in Epidemiology (STROBE) guidelines [9]. The study was approved by the Institutional Review Board of the Royal Thai Army Medical Department. Informed consent from each patient was waived owing to the retrospective design of the study.

### 2.2. Participants and Setting

**Figure 1:**
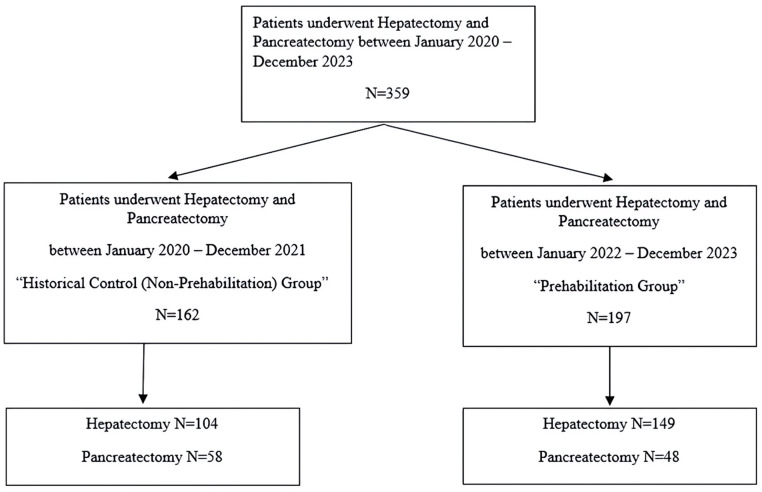
Flowchart of Participants and Setting. Comparing the Historical Control Group (2020–2021) and Prehabilitation Group (2022–2023).

Data was collected from the electronic medical records of patients who had undergone hepatectomy and pancreatectomy between January 2020 and December 2023. A standardized multimodal prehabilitation program was implemented institution-wide in January 2022. Consequently, the study compared two cohorts: a prehabilitation group (patients operated on between January 2022 and December 2023) and a historical control (non-prehabilitation) group (patients operated on between January 2020 and December 2021) (shown in Figure 1). Since patients in the historical group rarely underwent laparoscopic surgery, the prehabilitation group excluded laparoscopic procedures.

**Figure 2:**
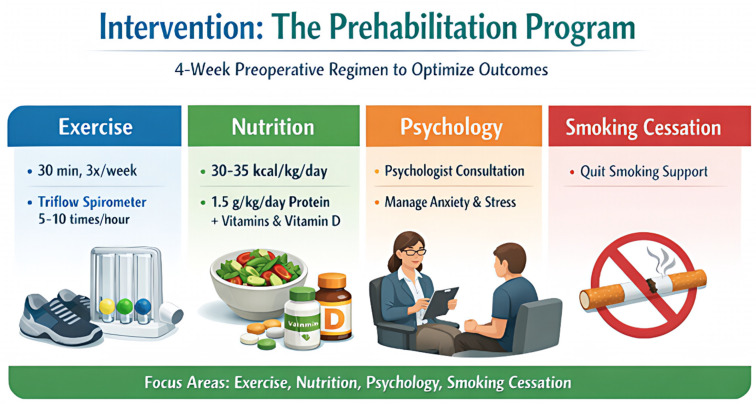
The Multimodal Prehabilitation Program.

The multimodal prehabilitation programs (shown in Figure 2).

*Intervention*: The Prehabilitation Program.

The prehabilitation protocol consisted of a 4-week preoperative regimen focusing on four pillars: exercise, nutrition, psychological support, and smoking cessation:

*Exercise:* Unsupervised moderate aerobic exercise (30 min/day, 3 times/week) and respiratory training using a triflow spirometer (5–10 times/h).

*Nutrition*: Dietary optimization guided by a nutritionist, targeting 30–35 kcal/kg/day and 1.5 g/kg/day of protein, supplemented with multivitamins and Vitamin D.

*Psychology*: Mandatory consultation with a psychologist to manage anxiety and stress.

*Postoperative Care*: All patients in the prehabilitation group were managed under an Enhanced Recovery After Surgery (ERAS) pathway [10,11] (shown in Figure 2).

To ensure compliance and maximize the efficacy of the intervention, patient adherence to the multimodal prehabilitation program was closely monitored. This was achieved through self-reported daily logbooks and weekly telephone follow-ups conducted by a dedicated clinical prehabilitation coordinator.

## 3. Outcomes and Statistical Analysis

The primary outcomes assessed were perioperative complications—classified according to the Clavien–Dindo system [12]—and 90-day mortality. Continuous variables were analyzed using either Student’s *t*-test or the Mann–Whitney U test and are presented as mean ± standard deviation (SD) or median with interquartile range (IQR). Categorical variables were compared using the Chi-square test or Fisher’s exact test and are reported as counts (percentages). All statistical analyses were conducted using STATA/IC 14.0 (StataCorp), considering a two-sided *p* value of <0.05 as statistically significant.

**Variables and Definitions** Baseline demographic and clinical variables included age, sex, body mass index (BMI) calculated as weight in kilograms divided by the square of height in meters (kg/m^2^) [13], American Society of Anesthesiologists (ASA) physical status [14], and preoperative serum albumin levels. Operative variables included the type of surgery, operative time, and estimated blood loss. To accurately assess outcomes based on surgical magnitude, procedures were stratified. Major hepatectomy was defined as the resection of three or more Couinaud segments, whereas minor hepatectomy involved the resection of fewer than three segments. Pancreatic resections were explicitly categorized into pancreatoduodenectomy (PD) and left pancreatectomy (LP) [15]. Postoperative morbidities were graded according to the Clavien–Dindo classification system, with major morbidity strictly defined as grade III or higher [12]. Additionally, subgroups for hepatectomy and pancreatectomy were created within both the control and prehabilitation groups.

### 3.1. Perioperative Outcomes in Hepatectomy

To accurately reflect the varying degrees of surgical stress, we analyzed the hepatectomy outcomes by separating major and minor resections. The indication for blood transfusion is determined by anesthesiology protocols, which specify transfusion when intraoperative blood loss exceeds 30 mL per kilogram of body weight or when hematocrit levels decrease to less than 30%. Throughout this study, the indications for perioperative blood transfusion were strictly determined by standard institutional anesthesiology protocols, which remained unchanged and were equally applied across both the historical control (2020–2021) and prehabilitation (2022–2023) periods.

### 3.2. Perioperative Outcomes in Pancreatectomy

Recognizing the different biological impacts of pancreatic procedures, we evaluated pancreatoduodenectomy (PD) and left pancreatectomy (LP) separately.

## 4. Results

### 4.1. Baseline Demographics and Nutritional Optimization

This study included a total of 359 patients: 162 in the historical control group and 197 in the prehabilitation group. Both cohorts were well-matched, with no significant differences in baseline characteristics such as age, gender, and body mass index (BMI). The most notable preoperative difference was in the patients’ nutritional status. Following the 4-week prehabilitation program, patients in the intervention group showed significant nutritional improvement, presenting with higher serum albumin levels on the day before surgery compared to the control group (*p* < 0.001) (shown in Table 1).

### 4.2. Perioperative Outcomes in Hepatectomy

In the major hepatectomy subgroup (control *n* = 30, prehab *n* = 40), the prehabilitation cohort trended toward shorter operative times and reduced blood loss, although these improvements did not reach statistical significance. Recovery metrics, including ICU and hospital length of stay, as well as complication rates, were similar between the two groups. However, a distinct clinical benefit was observed in the minor hepatectomy subgroup (control *n* = 74, prehab *n* = 109). Patients who completed the prehabilitation program required significantly fewer perioperative blood transfusions (8.3% vs. 18.9%, *p* = 0.033). Other outcomes, including hospital length of stay and overall morbidities, remained comparable (shown in Table 2).

### 4.3. Perioperative Outcomes in Pancreatectomy

The results for the PD subgroup (control *n* = 43, prehab *n* = 33) were highly encouraging. Patients in the prehabilitation group experienced shorter operative times (median 480 vs. 540 min, *p* = 0.014) and a shorter hospital stay, dropping from a median of 13 days down to 8 days (*p* < 0.001).

Benefits were equally pronounced in the LP subgroup (control *n* = 14, prehab *n* = 14). The prehabilitation cohort had significantly less blood loss (median 200 vs. 600 mL, *p* = 0.002), shorter ICU admissions (*p* = 0.011), and a faster discharge (median 5 vs. 9 days, *p* = 0.001).

There was no statistically significant difference in the 90-day mortality rate between the control and prehabilitation groups, including both the hepatectomy and pancreatectomy subgroups (shown in Table 3).

## 5. Discussion

This study demonstrates that a multimodal prehabilitation program is associated with improved perioperative outcomes in patients undergoing major HPB surgery. We observed significant reductions in operative time, blood loss, length of hospital stays, and overall morbidity following the implementation of our institutional protocol.

The surgical management of hepatopancreatobiliary (HPB) malignancies, specifically through hepatectomy and pancreatectomy, represents some of the most physiologically demanding procedures in modern medicine. As the patient demographic for these surgeries increasingly includes older and frailer individuals, the concept of prehabilitation—a multimodal intervention involving exercise, nutritional optimization, and psychological support—has gained prominence. While prehabilitation offers significant clinical benefits by enhancing physiological reserve, it also presents logistical and oncological challenges that must be critically evaluated.

The present study demonstrates that the implementation of a multimodal prehabilitation program yields varying degrees of clinical benefit depending on the magnitude of the hepatopancreatobiliary (HPB) resection. By stratifying our analysis into distinct procedural subgroups, we found that prehabilitation offers the most pronounced advantages for patients undergoing highly stressful, major operations.

Consistent with the recent literature, our most striking finding was observed in the pancreatoduodenectomy (PD) subgroup, where patients in the prehabilitation cohort experienced a significant reduction in the median hospital length of stay (from 13 to 8 days, *p* < 0.001). Left pancreatectomy (LP) patients similarly benefited from a shortened hospital stay and a decreased need for ICU admission. Interestingly, while the overall rate of minor morbidities was higher in the prehabilitation groups for pancreatic surgeries, the incidence of major morbidities (Clavien–Dindo ≥ 3) remained stable or trended downward. This suggests that prehabilitation, combined with Enhanced Recovery After Surgery (ERAS) pathways, may not completely prevent minor complications but plays a critical role in mitigating their severity and accelerating functional recovery, allowing for patients to be discharged sooner.

Conversely, the clinical benefits were less overt in the hepatectomy cohorts. While patients undergoing minor hepatectomies required significantly fewer perioperative blood transfusions, other postoperative parameters, including length of hospital stay, were not significantly altered by the 4-week program. This aligns with emerging evidence suggesting that the physiological buffer built by intensive prehabilitation is most crucial for high-stress procedures, whereas patients undergoing minor resections may naturally possess sufficient reserve to withstand the surgical trauma. Consequently, this highlights the necessity of risk-stratified prehabilitation pathways, ensuring that resources are prioritized for high-risk individuals or those facing complex, major resections.

The primary argument for implementing prehabilitation is the mitigation of postoperative morbidity through the improvement of “functional reserve”. Patients with pancreatic and liver cancers often present with malnutrition and sarcopenia, both of which are independent predictors of poor surgical outcomes. A landmark randomized clinical trial by Dunne et al. [16] demonstrated that a four-week structured exercise program significantly improved aerobic capacity in patients undergoing liver resection. Consequently, these patients exhibited a greater resilience to surgical stress, leading to reduced postoperative complications and shorter hospital stays. Furthermore, prehabilitation empowers patients, transforming them from passive recipients of care into active participants, which can significantly alleviate preoperative anxiety and improve overall quality of life.

However, the implementation of prehabilitation is not without significant disadvantages. The most pressing concern regarding hepatectomy and pancreatectomy is the delay to surgery. In the context of aggressive malignancies such as pancreatic ductal adenocarcinoma, the window for curative resection is narrow. There is a theoretical concern that delaying surgery by four to six weeks to accommodate a prehabilitation program could allow for tumor progression or metastasis. Although a systematic review by Luther et al. [17] suggests that a short delay for prehabilitation does not negatively impact oncological survival, the anxiety regarding tumor growth remains a significant psychological burden for patients and clinicians alike.

Despite the clinical benefits of prehabilitation being increasingly recognized, rigorous statistical validation remains a priority for the surgical community, in order to achieve standardized care. Dagorno et al. [18] conducted a critical systematic review and meta-analysis published in the *Journal of Visceral Surgery* to address the heterogeneity in current research. Their analysis confirmed that prehabilitation significantly reduces overall morbidity, specifically pulmonary complications, in hepatopancreatobiliary (HPB) surgery. However, they notably emphasized the need for higher-quality evidence, providing specific sample size calculations to guide future randomized controlled trials. This work underscores that while the current argument for the benefits of prehabilitation is strong, powering future studies correctly is a “necessary step forward” to firmly establish these protocols as the undisputed standard of care.

In the specific context of pancreaticoduodenectomy, the integration of nutritional support with physical therapy appears to be particularly effective. Tsukagoshi et al. [19] demonstrated that a combined preoperative strategy of rehabilitation and nutritional support could mitigate the impact of sarcopenia, a common condition in pancreatic cancer patients that severely compromises recovery. Their findings suggest that optimizing nutritional status is a prerequisite for effective physical rehabilitation; without it, exercise alone may not yield the desired physiological resilience required for such invasive procedures.

Finally, prehabilitation must be viewed as the foundational step in the broader continuum of “accelerated rehabilitation” and Enhanced Recovery After Surgery (ERAS). Chu et al. [20] observed that accelerated rehabilitation protocols significantly improve the clinical prognosis of patients undergoing pancreaticoduodenectomy, suggesting that preoperative preparation is essential for enabling this rapid postoperative recovery. This holistic approach is currently driving new research frontiers, as evidenced by Yao et al. [21]. Their recently published protocol for a randomized controlled trial highlights the evolving focus on integrating postoperative rehabilitation based on ERAS concepts, reinforcing the idea that surgical success depends on a seamless transition from preoperative optimization to postoperative reconditioning.

A primary concern in oncologic prehabilitation is the potential delay to surgery. However, our program was integrated without compromising oncologic safety, aligning with systematic reviews suggesting that the benefits of physical optimization outweigh the theoretical risks of short delays.

Our stratified findings align with recent studies indicating that the clinical benefits of prehabilitation are most pronounced in patients undergoing highly stressful, major resections. Similar to findings reported by recent ERAS and prehabilitation trials, we observed a significant reduction in the length of hospital stay among patients undergoing pancreatoduodenectomy. This suggests that prehabilitation effectively optimizes functional reserve, allowing patients to recover faster from profound surgical trauma. Conversely, the less pronounced impact observed in the minor hepatectomy subgroup mirrors emerging evidence that routine, intensive 4-week prehabilitation protocols might not yield significant outcome differences for lower-stress procedures. This highlights a critical need for risk-stratified prehabilitation pathways, where resources are prioritized for high-risk patients or those facing major, technically demanding resections where the physiological buffer is most challenged.

Interestingly, we observed improvements in intraoperative factors such as blood loss and operative time. While prehabilitation primarily targets patient physiology, these findings may reflect the synergistic effect of the ERAS pathway applied postoperatively in the intervention group, or potentially better patient compliance and physiological tolerance during anesthesia.

## 6. Limitations

We acknowledge several limitations in our study. First, the retrospective, historical control design introduces inherent temporal biases. In our initial analysis, we observed trends toward shorter operative times and reduced estimated blood loss in the prehabilitation cohort. However, we must interpret these intraoperative metrics with caution. There is no established biological mechanism by which physical prehabilitation independently reduces surgical bleeding. Instead, these intraoperative improvements are highly likely to be attributable to the natural evolution of surgical practice at our institution over the study periods (2020–2021 vs. 2022–2023), including institutional learning curves, and the introduction of advanced surgical technologies. Future large-scale, randomized controlled trials that stratify patients by surgical magnitude and strictly control for advancements in surgical techniques are warranted to validate our findings. In addition, since the historical group rarely carried out laparoscopic surgery, the prehabilitation group excluded laparoscopic procedures.

## 7. Conclusions

The implementation of a multimodal prehabilitation program is associated with significantly reduced postoperative morbidity, shorter hospital stays, and improved intraoperative metrics in patients undergoing major HPB surgery. These findings support the integration of prehabilitation into standard surgical pathways to optimize recovery.

## Figures and Tables

**Table 1:** Demographic data.

	Non-Prehabilitation Group (*n* = 162)	Prehabilitation Group (*n* = 197)	*p*-Value	Hepatectomy Non-Prehabilitation (*n* = 104)	Hepatectomy Prehabilitation (*n* = 149)	*p*-Value	Pancreatectomy Non-Prehabilitation (*n* = 58)	Pancreatectomy Prehabilitation (*n* = 48)	*p*-Value
Age (year)	59.99 ± 13.7	60.81 ± 13.19	0.566	60.47 ± 12.36	60.68 ± 12.17	0.895	59.14 ± 15.9	61.23 ± 16.09	0.504
Male	102 (63%)	134 (68%)	0.364	76 (73.1%)	110 (73.8%)	0.894	26 (44.8%)	24 (50%)	0.595
Body weight (kg)	65.41 ± 12.41	65.01 ± 15.3	0.785	66.84 ± 12.77	65.7 ± 15.46	0.537	62.85 ± 11.4	62.83 ± 14.71	0.996
Height (cm)	162.88 ± 7.7	164.21 ± 7.96	0.110	163.39 ± 7.99	165.19 ± 7.71	0.073	161.95 ± 7.11	161.15 ± 8	0.586
BMI (kg/m^2^)	24.63 ± 4.36	24.07 ± 5.29	0.280	25.03 ± 4.6	24.03 ± 5.18	0.114	23.92 ± 3.84	24.21 ± 5.68	0.768
Serum albumin at diagnosis (g/mL)	4.01 ± 0.48	4.09 ± 0.4	0.086	4.04 ± 0.45	4.1 ± 0.4	0.261	3.96 ± 0.53	4.07 ± 0.38	0.247
Serum albumin before surgery (g/mL)	3.79 ± 0.5	4.17 ± 0.42	<0.001 *	3.76 ± 0.49	4.16 ± 0.44	<0.001 *	3.86 ± 0.5	4.18 ± 0.38	<0.001 *
Albumin difference (g/mL)	−0.21 ± 0.5	0.08 ± 0.35	<0.001 *	−0.28 ± 0.52	0.06 ± 0.36	<0.001 *	−0.1 ± 0.43	0.12 ± 0.33	0.004 *
ASA									
0	47 (29%)	53 (26.9%)	<0.001 *	3 (2.9%)	16 (10.7%)	0.017 *	44 (75.9%)	37 (77.1%)	0.883
1	106 (65.4%)	139 (70.6%)		92 (88.5%)	128 (85.9%)		14 (24.1%)	11 (22.9%)	
2	9 (5.6%)	5 (2.5%)		9 (8.7%)	5 (3.4%)				

Independent *t* test and Chi-square test. * statistically significant.

**Table 2:** Hepatectomies.

	Hepatectomy Non-Prehabilitation (*n* = 104)	Hepatectomy Prehabilitation (*n* = 149)	*p*-Value	Major Hepatectomy Non-Prehabilitation (*n* = 30)	Major Hepatectomy Prehabilitation (*n* = 40)	*p*-Value	Minor Hepatectomy Non-Prehabilitation (*n* = 74)	Minor Hepatectomy Prehabilitation (*n* = 109)	*p*-Value
Operative time (min)	240 (200, 360)	250 (210, 300)	0.828	360 (277.5, 420)	3350 (240, 390)	0.254	240 (180, 300)	240 (200, 300)	0.375
Blood loss (mL)	500 (250, 1000)	400 (200, 700)	0.101	925 (600, 1600)	700 (400, 1470)	0.210	350 (200, 700)	300 (200, 500)	0.316
Need for blood transfusion	28 (26.9%)	24 (16.1%)	0.036 *	14 (46.7%)	15 (37.5%)	0.441	14 (18.9%)	9 (8.3%)	0.033 *
ICU stay (days)	2 (1, 3)	2 (0, 2)	0.462	3 (2, 3)	2 (2, 3)	0.807	2 (0, 2)	2 (0, 2)	0.629
Hospital stay (days)	7.5 (6, 9)	6 (5, 9)	0.042 *	9 (8, 12)	9 (6, 14)	0.365	6.5 (5, 9)	6 (5, 8)	0.117
Overall morbidities	33 (31.73%)	20 (13.42%)	0.222	9 (30%)	12 (35%)	0.659	11 (14.9%)	19 (17.4%)	0.645
Major morbidities (Clavien–Dindo ≥ 3)	10 (30.3%)	5 (25%)	0.542	5 (16.7%)	8 (20%)	0.723	0	2 (1.8%)	0.516
90-day mortality, yes	1 (1%)	4 (2.7%)	0.333	1 (3.3%)	3 (7.5%)	0.630	0	1 (0.9%)	1.000

Mann–Whitney U test, Chi-square test, and Fisher’s exact test, * statistically significant.

**Table 3:** Pancreatectomies.

	Pancreatectomy Non-Prehabilitation (*n* = 58)	Pancreatectomy Prehabilitation (*n* = 48)	*p*-Value	PD Non-Prehabilitation (*n* = 43)	PD Prehabilitation (*n* = 33)	*p*-Value	LP Non-Prehabilitation (*n* = 14)	LP Prehabilitation (*n* = 14)	*p*-Value
Operative time (min)	510 (420, 600)	480 (360, 540)	0.048 *	540 (480, 630)	480 (420, 540)	0.014 *	360 (255, 465)	300 (300, 360)	0.589
Blood loss (mL)	600 (400, 1000)	425 (225, 725)	0.008 *	600 (450, 1100)	600 (300, 850)	0.219	600 (462.5, 800)	200 (162.5, 400)	0.002 *
Need for blood transfusion	26 (44.8%)	20 (41.7%)	0.744	23 (53.3%)	17 (51.5%)	0.864	3 (21.4%)	2 (14.3%)	1.000
ICU stay (days)	3 (2, 3)	2 (2, 3)	0.1	3 (2, 3.5)	3 (2, 3)	0.619	2 (0.5, 3)	0 (0, 0.75)	0.011
Hospital stay (days)	13 (10, 15)	7 (6, 10)	<0.001 *	13 (11.5, 15)	8 (7, 10)	<0.001 *	9 (7.5, 13.5)	5 (4.25, 6)	0.001 *
Overall morbidities	43 (74.13%)	30 (62.5%)	0.650	29 (67.4%)	20 (60.6%)	0.55	8 (57.1%)	10 (71.4%)	0.822
Major morbidities (Clavien-Dindo ≥ 3)	9 (20.93%)	3 (7.89%)	0.017 *	7 (16.3%)	3 (9.1%)	0.499	2 (14.3)	0	0.481
Mortality, yes	2 (3.4%)	2 (4.2%)	0.847	2 (4.7%)	2 (6.1%)	1	0	0	1.000

Mann–Whitney U test, Chi-square test, and Fisher’s exact test, * statistically significant.

## Data Availability

The datasets generated and/or analyzed during the current study are available from the corresponding author on reasonable request.
